# Facilitators and barriers to Latino/a adolescents' help‐seeking behaviors: Discussing mental health needs with parents and engaging in mental health services

**DOI:** 10.1111/jora.70232

**Published:** 2026-07-24

**Authors:** Fernanda Lima Cross, Joel Lucio, Amanda Webster, Zachary Sessa, Irving Suarez, Kenneth Resnicow

**Affiliations:** ^1^ School of Social Work University of Michigan Ann Arbor Michigan USA; ^2^ School of Public Health University of Minnesota Minneapolis Minnesota USA

**Keywords:** access to care, conversations about mental health, Latino adolescents, Latino parents, mental health, mental health literacy, mental health stigma

## Abstract

Latino/a adolescents report worse mental health than other ethnic–racial groups in the United States, including elevated depressive symptoms and anxiety, yet they remain less likely to access mental health services. Negative perception about mental health within Latino/a communities, shaped by negative beliefs about mental illness and limited knowledge of treatment, poses significant obstacles to care. Low mental health literacy, including poor understanding of depression, anxiety, and the role of therapy and medication, further contributes to negative attitudes about mental health. Prior work shows that Latino/a adolescents often avoid disclosing mental health concerns to parents to prevent burdening them, and this lack of communication impacts their help‐seeking behaviors. To examine these challenges, we conducted six focus groups (*n* = 56) with Latino/a parents and adolescents aged 14–17 from Michigan and Texas. Analysis identified negative perceptions about mental health as a central obstacle for both parents and adolescents, limiting open dialogue about mental health. Adolescents reported fears of judgment or symptom minimization, contributing to delayed help‐seeking behaviors. Parents expressed a need for greater mental health knowledge, including symptom recognition, treatments, and strategies for discussing mental health with their adolescent children. Adolescents emphasized the importance of trust, confidentiality, and family acceptance in facilitating disclosure and support. Findings underscore the need for interventions that reduce negative perception about mental health, improve mental health literacy, and strengthen parent–adolescent communication to enhance Latino/a adolescents' access to mental health care. Practitioners can support families by providing tools that support dialogue and reduce negative‐perceptions about mental health.

Latinos/as in the United States comprise nearly one‐fifth of the country's population, and continue to represent one of the largest and fastest‐growing adolescent populations (U.S. Census Bureau, 2024). Approximately 32% of Latino/a individuals in the United States are under the age of 18, making Latino/as the nation's youngest ethnic‐racial group (Piña & Martinez, 2025). Adolescence is a transformative developmental period with challenges that can impact mental health; however, Latino/a adolescents face added vulnerabilities from experiences such as discrimination and acculturative or bicultural stress (McCord et al., 2019; Suppiej et al., 2025; World Health Organization, 2025). Consequently, Latino/a adolescents experience elevated risk of depression and anxiety compared to White adolescent counterparts (Borrero & Przeworski, 2025). Despite growing concerns about mental health resources, Latino/a adolescents are less likely to engage in assessment, diagnosis, and treatment for mental health issues compared to other groups (Borrero & Przeworski, 2025; Hoffmann et al., 2022).

This service gap is especially consequential given disproportionate mental health disparities in the Latino/a population, including lower rates of mental health service utilization despite similar or higher rates of mental health conditions compared to other ethnic groups (Brewer et al., 2024). Barriers to care include structural constraints (e.g., language barriers, financial disparity, lack of culturally responsive providers, time availability) as well as barriers that shape whether adolescents' needs are recognized and discussed within families. In particular, negative perceptions of mental health and low mental health literacy may reduce the likelihood that adolescents discuss mental health concerns with caregivers, thus influencing help‐seeking behaviors and treatment engagement (Henderson et al., 2013; Pérez‐Flores & Cabassa, 2021).

Negative perceptions about mental health refer to micro to macro level social attributions of stereotype or bias that shape attitudes and experiences related to mental health (Rössler, 2016; Thornicroft, 2006). This includes cultural norms (general population's endorsement of mental health stereotype), the self (when oneself internalizes publicly endorsed stereotype), and the structural (in which policy reinforces the bias through barriers to mental health care) (Corrigan et al., 2014). Mental health literacy refers to knowledge and beliefs about the causes, treatment, and trajectory of mental illness (Jorm et al., 1997; Sampaio et al., 2022). Cross‐sectional studies on mental health literacy find that higher self‐report levels of mental health literacy are associated with a greater likelihood of treatment seeking and treatment compliance (Devi et al., 2025).

Mental health literacy intersects with negative attitudes and perceptions about mental health, creating complex barriers to parent‐adolescent communication (Dixon De Silva et al., 2020; Pérez‐Flores & Cabassa, 2021). Literature further suggests that Latino/a adolescents and parents have lower mental health literacy, higher negative perceptions of mental health, and lower mental health care use (Benuto et al., 2019; Dixon De Silva et al., 2020). Understanding how mental health literacy and negative perceptions jointly impact parent‐adolescent conversations is essential for developing effective interventions and improving mental health outcomes in these communities.

## Theoretical framework

In this study, we utilize the Disclosure Decision‐Making Model (DD‐MM) to guide our understanding of how negative attitudes toward mental illness play a role in mental health conversations between adolescents and their parents, as well as how such attitudes influence help‐seeking behaviors. The DD‐MM model has been shown to be a useful framework that conceptualizes the various factors involved in disclosing personal health information, including diagnoses or health issues that potentially face negative attitudes (Greene, 2009). This has been supported across interpersonal and motivational contexts, including severe mental health disclosure with family (Greene et al., 2012; Pahwa et al., 2017). The DD‐MM model outlines a dynamic process where, at any given point, people consider the risks and benefits of intentionally sharing personal health information. If a person decides to move forward after an assessment, they follow the next phase of the disclosure process.

There are three primary phases to the DD‐MM model where a person makes an assessment to ultimately disclose–assessing the information, assessing the receiver, and assessing disclosure efficacy. In each level of assessment, a person considers particular factors in their disclosure‐making process. In the first phase, information assessment (IA), a person weighs their knowledge or perception of a diagnosis or health problem, including negative health perceptions and symptom severity (Greene, 2009). As such, internalized negative perceptions of their health diagnosis, poor health literacy, or mild symptoms are associated with a reduced likelihood of disclosure and help‐seeking behaviors (Benuto et al., 2019; Chaudoir & Fisher, 2010; Pahwa et al., 2017). In the second phase, receiver assessment (RA), a person considers to whom they may disclose their health information, as well as the relational quality, anticipated response, and anticipated outcome. For example, close family and friendships increase the possibility of disclosure, whereas weak or strained family or friendships make disclosing less likely (Pahwa et al., 2017). Notably, whomever is chosen to receive health information is dependent on the discloser's perception of social support (Pahwa et al., 2017). Finally, in the third phase, disclosure efficacy assessment (DEA), a person considers one's disclosure efficacy, or their perceived ability to share health information with specified people for desired outcomes (Greene, 2009). Increased efficacy is associated with greater intention to disclose health information with others across contexts (Greene et al., 2012). On the other hand, negative perceptions about mental health, anticipated negative responses, or depressive symptoms may lead to avoidance of emotional topics or reduced efficacy (Carpenter & Theiss, 2023).

Ultimately, the kind of health information being considered for disclosure may impact the disclosure process itself (Greene, 2009). For example, the disclosure process may appear different for someone diagnosed with HIV than for someone with a broken arm. In a study by Rasmussen et al. (2022), this differentiation has been shown to be apparent for adolescents experiencing mental health problems, with empathy being a factor greatly considered in the disclosure process. Moreover, the study suggests that when an adolescent has a mental health disorder, or when parents contribute to the disorder itself, it may alter their perception of disclosure (Rasmussen et al., 2022). It is important to note, however, that the participants in this study mostly identified as White (non‐Hispanic), and therefore their findings on adolescents' disclosure process may not be representative to other groups such as Latinos/as.

## Developmental risk factors

Adolescence is an important developmental period characterized by heightened vulnerability to mental health concerns due to rapid physical, biological, emotional, and social changes that can affect mental health (World Health Organization, 2025). While experiencing body maturation, identity exploration, and changing relational dynamics, adolescents also undergo novel stressors that can lead to increased risk for mental health challenges, including navigating social media influence, peer relationships, school pressures, and emotional fluctuations (Suppiej et al., 2025). Latino/a adolescents, in particular, face added vulnerabilities from unique challenges such as disproportionate exposure to violence, ethnic‐racial discrimination, acculturative or bicultural stress, and social exclusion. These experiences can further contribute to internalizing and externalizing symptoms (Santacrose et al., 2021; McCord et al., 2019). Additionally, Latino/a adolescents are at greater risk of living in poverty and have difficulties accessing mental health care (Pérez‐Flores & Cabassa, 2021). Due to these intersecting developmental and sociocultural factors, Latino/a adolescents are vulnerable to experiencing mental health challenges, which further underscores the need of understanding the factors that shape their help‐seeking behaviors.

## Mental health literacy in Latino/a families

Mental health literacy is an important factor that impacts how families discuss and address mental health concerns. Mental health literacy refers to knowledge and beliefs about the causes, treatment, and trajectory of mental illness (Jorm et al., 1997; Sampaio et al., 2022). Greater mental health literacy includes symptom recognition, knowledge of available treatment options, and subsequent help‐seeking behaviors, such as engaging in mental health services. For adolescents, mental health literacy is evaluated through similar means to adults (symptom recognition, knowledge of available treatment); however, while service engagement is developmentally appropriate, it is within the social constraints of adolescence. Adolescents may not have access to the full scope of mental health service options due to legal, financial, or logistical barriers. Instead, they rely on mental health help‐seeking such as reaching out to a school counselor, identifying with and disclosing concerns with a trusted adult, or navigating constrained services options (particularly older adolescents). Within Latino/a families, mental health literacy varies significantly based on educational attainment, language access, and exposure to mental health services. Depression knowledge, for instance, was significantly higher among Hispanic women with some college education versus those with lower education levels (Brewer et al., 2024). This educational difference affects families' ability to recognize mental health symptoms, understand treatment options, and engage in meaningful conversations about mental health with their children.

Latino/a patients often enter mental health treatment through primary care settings, making these environments critical for screening and education (Brewer et al., 2024). However, Latino/a adolescents and families are at higher risk for premature dropout and poor engagement in mental health services for adolescents, reflecting gaps in literacy and access (Nogueira & Schmidt, 2022). Communities report significant shortages of bilingual family/child therapists and culturally accessible services, limiting caregivers' ability to seek appropriate care (Tien, 2023). These structural barriers compound the effects of low mental health literacy by reducing opportunities for families to gain knowledge through service engagement.

Understanding of mental health issues plays a significant role in shaping attitudes and treatment‐seeking behaviors (DeFreitas, 2022). Among some Latinos/as, misconceptions about depression stem from attributions to supernatural causes or moral failings (Caplan et al., 2011). Enhanced comprehension of the neurobiological foundations of mental health conditions is associated with more favorable attitudes toward mental health and treatment. However, the relationship between knowledge and attitudes demonstrates complexity within Latino/a populations. In a sample of *n* = 350 Hispanic adults seeking primary care treatment for depression, the authors found that when participants received culturally adapted psychoeducational material, they saw a reduction in depressive symptoms and self‐reported measures of stigma, while also seeing an increase in mental health literacy. Among Latino/a clients with depression, many reported increased medication concerns as their knowledge expanded, possibly due to greater awareness of adverse effects (Sanchez et al., 2019). Culturally responsive and developmentally appropriate mental health education is therefore crucial for Latino/a communities.

## Prevalence and nature of negative perceptions about mental health

Negative attitudes and perceptions about mental health are widespread within Latino/a populations and create substantial obstacles to accessing care and discussing mental health within families. Evidence from a study of Spanish‐speaking Latino/a individuals experiencing depression revealed that the vast majority, 83%, held negative perceptions of mental health (Collado et al., 2019). Similarly, among a diverse sample of Asian‐American and Latino/a‐American adolescent students, 86.4% identified negative attitudes as an impediment to pursuing treatment (Wang et al., 2019). These negative attitudes are embedded in cultural beliefs and social dynamics. Parents and community members report that seeking mental health help can make a child an outsider or make a parent feel like a failure, which fuels secrecy and avoidance (Bismar, 2018). Concerns about antidepressant use and therapy are prevalent among Hispanic patients and vary by education level, with those with higher levels of education more likely to engage in treatment (Brewer et al., 2024). Among Latino/a adolescents receiving psychiatric services, nearly all participants described experiencing shame associated with their diagnoses, which subsequently diminished their sense of self‐worth (Elkington et al., 2012). Parents from Latino/a backgrounds have reported apprehension that seeking treatment for children experiencing emotional distress would result in judgment from their broader social networks, including extended family and community members (Dixon De Silva et al., 2020).

Additionally, parents have voiced concerns that pursuing mental health services for their children could trigger adverse consequences such as special education placement or future employment obstacles (Chavira et al., 2017). Some parents have also expressed fears regarding potential loss of custody rights (Dixon De Silva et al., 2020). In the United States, public campaigns to reduce negative attitudes about mental health and clinical messaging have predominantly been disseminated through English media, leaving Spanish‐speaking and Latino/a media consumers less reached by corrective messages (Quezada & Fuentes, 2025). This information gap perpetuates misconceptions and negative attitudes within Latino/a communities.

## Impact on parent–adolescent mental health conversations

To explore barriers and facilitators of Latino/a adolescents' help‐seeking behaviors, particularly their willingness to discuss mental health needs with parents and engage in mental health services, parent‐adolescent communication must be considered. Within the DD‐MM framework outlined above, the likelihood of disclosing mental health concerns increases when anticipated benefits outweigh the perceived barriers and risks (Greene, 2009). Therefore, we conceptualize parent‐adolescent communication as a critical, early step in the help‐seeking process that can either facilitate or hinder Latino/a adolescents' service utilization.

There is scant literature examining parent–adolescent mental health conversations within Latino/a families. Based on the studies available, it is known that the intersection of low mental health literacy and negative perceptions of mental health impacts communication regarding mental health within Latino/a families. Negative attitudes about mental illness decrease parent–child communication about personal mental health, with reduced communication mediating worse help‐seeking attitudes and behaviors among adolescents with immigrant parents. Parental negative beliefs of mental health discourage help‐seeking and limit parents' identification of child anxiety and depression, as well as their initiation of treatment pathways (Bismar, 2018). Parents serve as crucial gatekeepers to mental health services, and their attitudes significantly influence whether adolescents receive needed care. Differences in acculturation between parents and adolescents can moderate how negative attitudes affect willingness to talk about and seek help for mental health issues. This dynamic is particularly relevant in Latino/a families where generational differences in cultural adaptation are common. Additionally, Latino/a individuals at risk for depression who anticipate negative reactions are substantially less likely to share their diagnostic information with relatives or friends (Vega et al., 2010).

## Financial strain and access to health services

Access to mental health services for Latino/a families is constrained by intersecting financial and socioeconomic barriers that force difficult choices between immediate survival needs and healthcare. Among Latino/a adults with unmet mental health needs, an estimated 40% cited the cost of treatment as prohibitive. Approximately 10.2 million Hispanics in the United States lack health insurance coverage, representing a higher uninsured rate compared to other demographic groups (UnidosUS, n.d.; Parra‐Cardona & DeAndrea, 2016). This lack of insurance translates directly into reduced service utilization as Latino/a adults were 28% less likely than other adults to receive mental health treatment in 2024 (U.S. Department of Health and Human Services, Office of Minority Health, 2025). For many low‐income Latino/a families, these financial constraints intersect with the prioritization of basic survival needs over healthcare, creating a hierarchy of necessities in which mental health services are a low priority.

Cabassa et al. (2006) highlight how economic strain, such as an inability to pay for food, rent, and other basic necessities, is negatively associated with mental health service utilization. Latino/a families in poverty face trade‐offs between healthcare and immediate needs as paying for a doctor's visit can mean being unable to pay for essentials like phone bills or gas. During the COVID‐19 pandemic, these dynamics intensified as low‐income Latino/a families experienced job losses and reduced work hours, with youth reporting families relying on food banks or churches and delaying rent payments to cover expenses, while parents continued working despite health risks due to economic necessity (Cortés‐García et al., 2022). For low‐income Latino/a families, mental health and healthcare are generally secondary priorities to immediate survival needs, with financial barriers and work obligations creating structural conditions in which families are forced by circumstances to choose economic stability over health.

## Cultural factors influencing mental health communication

### The role of Familismo in mental health help‐seeking

The cultural value of familismo plays a central role in Latino/a communities and shapes how mental health is perceived and discussed. Familismo refers to a cultural emphasis on loyalty, interconnectedness, and mutual obligation among immediate and extended family members, which, while generally protective of well‐being (Marín & Marín, 1991; Parsai et al., 2008; Santiago‐Rivera, 2003), can sometimes inhibit open discussion of mental health problems by encouraging privacy and avoidance of topics that might bring shame or burden to the family. Family orientation and concern about others' opinions increase social distance and secrecy around mental illness, affecting both adolescents' willingness to disclose and parents' readiness to accept formal care (Bismar, 2018; Nogueira & Schmidt, 2022). Family members may face blame for their relative's condition or become targets of fears about contagion (DeFreitas, 2022). Paradoxically, despite the centrality of family relationships, many Latino/a individuals show reluctance to communicate about their mental health struggles with family members due to concerns about negative reactions (DeFreitas, 2022). Evidence indicates that familismo also influences treatment choices, with individuals exhibiting strong familismo‐related behaviors showing a greater likelihood of pursuing informal support networks or faith‐based interventions rather than conventional medical or psychological treatment (Villatoro et al., 2014). Latino/a adolescents, particularly young men and those who perceive high levels of public negative attitudes, often anticipate that revealing their mental health struggles will lead to social rejection. When this potential rejection extends to family relationships, the risk of worsened mental health outcomes may drive many to maintain silence about their difficulties. This framework may intensify negative perceptions of mental health by positioning mental illness as incompatible with cultural ideals of family, strength, and resilience (DeFreitas, 2022).

## How service experiences reinforce negative mental health beliefs

Prior experience with mental health services can either amplify or diminish negative attitudes while simultaneously affecting mental health literacy. Latino/a clients, especially those born abroad, frequently face challenges including early discontinuation of services and receipt of substandard care (DeFreitas, 2022). When individuals have unfavorable treatment experiences, these encounters tend to strengthen negative beliefs and discourage future help‐seeking (Diala et al., 2000). Conversely, beneficial therapeutic experiences can help improve attitudes and knowledge. Research with Spanish‐speaking individuals experiencing depression found that those who participated in supportive counseling emphasizing emotional expression demonstrated greater reductions in negative attitudes compared to participants receiving behavioral activation interventions (Collado et al., 2019).

Harmful stereotypes portraying Latino/a individuals as particularly vulnerable to substance abuse may also contribute to heightened concerns surrounding psychiatric medications like antidepressants, while therapy faces less resistance (Interian et al., 2010). The misconception that antidepressant medications carry addiction risks (Vargas et al., 2015) generates resistance within Latino/a communities, where individuals are reluctant to be associated with any form of drug use, including legitimately prescribed psychotropic medications. Such experiences may intensify negative attitudes toward psychiatric medication and amplify concerns about facing discrimination related to having a mental health condition (DeFreitas, 2022).

## The current study

This study was grounded by the research questions: What are the facilitators and barriers to adolescents' (a) discussion of mental health needs with caregivers or other adults, and (b) use of mental health services? We conceptualize parent–adolescent communication as a critical early step in the help‐seeking process that may facilitate or hinder their engagement in services. To our knowledge, no studies have specifically examined Latino/a adolescents' mental health disclosure processes. Focusing on this population and context may provide important insight to inform culturally responsive interventions designed to strengthen help‐seeking behaviors, promote parent–adolescent discussions about mental health, and increase engagement with mental health services.

### Positionality of data analysis team members

I, Fernanda, am a Latina immigrant. I am the project's PI and was involved in all aspects of the study. As an immigrant and mother of adolescents, relating with participants who were also Latino/a immigrants felt natural, which facilitated rapport as well as data analysis and write up. Weekly team meetings were crucial for reducing biases and supporting analytical rigor.

I, Joel, am a third‐generation Mexican American. I related to all aspects of the project as both an insider and outsider. I share culture and language with many participants yet have not personally experienced particular structural challenges. Working as a mental health therapist provided greater in‐depth knowledge of the complexities in seeking mental health care. Any preconceived assumptions were independently acknowledged and discussed with coding team members to reduce bias in data interpretation.

I, Irving, am a first‐generation Mexican American and first‐generation college graduate with a Master of Public Health degree. My lived experiences facilitated cultural understanding, while my formal education shaped my interpretation of structural and systemic factors affecting the community. Throughout the research process, any preconceived assumptions were acknowledged and discussed with team members to reduce bias and strengthen analytic rigor.

I, Zach, critically reflected on the privileges attached to my identity as a white man with birthright citizenship in the United States because I do not share the cultural background nor lived experiences of the families. My work as a teacher and social worker with first‐generation Latino/a youth and families in the United States, México, and Chile shaped my awareness of barriers Latino/a families face in accessing and discussing mental health care.

I am Amanda, a white, queer, cisgender woman with birthright U.S. citizenship. My background in intergroup relations and social work informs my interest in how language, ethnic‐racial identity, and citizenship shape mental health experiences. Because my privileged identities differ from participants', I approach this research with humility, curiosity, and attention to positionality.

## METHOD

### Study overview

This community‐based participatory research was conceptualized in response to ongoing involvement with a community advisory board and previous interviews with Latino/a parents and adolescents where support for the mental health of adolescents was identified as a community need. In order to better understand the facilitators and barriers for mental health service access a total of 29 Latino/a parents and 27 adolescents (56 total individuals) participated in six focus groups conducted through the Zoom platform in 2025 (March–May). Participants resided in Michigan and Texas. Two focus groups included adolescents only (*N* = 17), two separate focus groups included parents only (*N* = 19), and two focus groups combined both parents and adolescents (*N* = 20). We conducted separate focus groups with parents and adolescents in order to learn about their different experiences and perspectives. The joint focus groups provided an opportunity for related parents and adolescents to exchange their perspectives and learn from each other's' experiences. Having been diagnosed with a mental health condition was not a requirement for participation in the study, but participants expressed some level of familiarity with mental health issues, either personally, through a family member, friend, colleague, or through the media.

A total of 31 parents filled out our anonymous demographic survey, and 29 parents participated in the focus groups. Among them, 27 were female and four were male with ages ranging from 30 to 59 and 77% being 40–49 years old. Thirty‐nine percent of participants had a high‐school diploma or less while 61% had some college or higher levels of education. About 58% of the sample was employed part or full time with the large majority (55%) earning $50,000 or less, while 29% earned between $50,000 and $100,000 and 16% did not respond. All participants were Latino/a immigrants coming from diverse South American countries and Mexico (68%) having arrived in the US between the ages of 10 and 49 with most arriving after age 20. A total of 28 adolescents filled out our survey and 27 participated in the focus groups. Fifteen were males and 13 were females ranging in ages from 14 to 18 and all of them were in high school. Fourteen percent of them worked part‐time and almost 90% of the adolescents aimed to obtain at least a college degree. Half of the adolescents (50%) were born in the US, 29% in Mexico (29%) and the others in different South American countries. Most of the adolescents born abroad arrived in the US after the age of 10. See Table 1 for the complete parents and adolescent demographics.

**TABLE 1:** Demographics table.

Variable	Parents		Adolescents	
	*n*	%	*n*	%
**Gender**				
Female	27	87	13	46
Male	4	13	15	54
Total	31	100	28	100
**Age**				
14	‐	‐	9	32
15	‐	‐	5	18
16	‐	‐	7	25
17	‐	‐	7	25
30–39	2	6	‐	‐
40–49	24	77	‐	‐
50–59	5	16	‐	‐
Total	31	100	28	100
**School grade**				
9th	‐	‐	11	39
10th	‐	‐	5	18
11th	‐	‐	8	29
12th	‐	‐	4	14
Total	‐	‐	28	100
**Education** ^a^				
Elementary school	1	3	‐	‐
Middle school	3	10	‐	‐
Some high school	2	6	‐	‐
High School/GED degree	6	19	1	4
Associate's degree	1	3	2	7
Some college	5	16	‐	‐
Bachelor's degree	7	23	12	43
Professional/Graduate degree	6	19	13	46
Total	31	100	28	100
**Employment status**				
Student	‐	‐	17	17
Full‐time	11	37	‐	‐
Part‐time	7	23	4	14
Unemployed	6	20	6	6
Other	5	17	‐	‐
Unable to work	1	3	1	4
Total	30	100	28	100
**Income**				
Less than $25,000	6	23	‐	‐
$25,000 to $50,000	11	42	‐	‐
$50,000 to $100,000	5	19	‐	‐
More than $100,000	4	15	‐	‐
Total	26	100	‐	‐
**Age of U.S. arrival**				
U.S. Born	‐	‐	10	43
Less than 10 Years Old	‐	‐	9	39
10–19	5	16	4	17
20–29	11	35	‐	‐
30–39	8	26	‐	‐
40–49	7	23	‐	‐
Total	31	100	23	100
**Migratory status**				
U.S. Citizen	2	14	8	67
Permanent Resident	1	7	2	17
Documented (with Valid Visa)	8	57	2	17
Undocumented	3	21	‐	‐
Total	14	100	12	100
**Country of birth**				
Bolivia	1	3	‐	‐
Brazil	‐	‐	1	4
Costa Rica	1	3	‐	‐
Dominican Republic	1	3	‐	‐
Honduras	‐	‐	1	4
Mexico	21	68	8	29
Peru	1	3	1	4
United States	‐	‐	16	57
Venezuela	6	19	1	4
Total	31	100	28	100
**Familial heritage**				
Bolivia	‐	‐	1	4
Brazil	‐	‐	1	4
Costa Rica	‐	‐	1	4
Honduran	‐	‐	1	4
Mexico	‐	‐	17	61
Mixed	‐	‐	3	11
Peru	‐	‐	1	4
Venezuela	‐	‐	3	11
Total	‐	‐	28	100

*Note*: *N =* 59 (*n* = 31 for Parents, *n* = 28 for Adolescents) for this table. However, 3 participants (two parents and one Adolescent) did not attend focus groups. A dash (−) indicates that the variable did not apply to the respondent. These responses are distinct from missing or skipped responses.

^*a*^ Education category differs between the “Parent” and “Adolescent” group. The “Parent” group was asked their highest level of education completed, while the “Adolescent” group was asked how much education they wish to complete.

Focus groups lasted about 90 min each and were audio recorded. Participants had the option of being interviewed in English or Spanish, and were grouped according to their preference. All parents chose to be interviewed in Spanish. Four focus groups were conducted in Spanish and two in English (with adolescents only). A team of three bilingual interviewers, which included the Principal Investigator (PI) and two graduate students, conducted the focus groups. To increase safety, participants were asked to choose pseudonyms and keep their cameras off. We also completely de‐identified the data and erased audio recordings after transcriptions were complete. This meant we were unable to fully track what participants had said throughout the focus groups by referencing the final transcripts alone. However, during the focus groups, some participants shared their pseudonym before speaking, or the interviewer referred to the participants by their pseudonym in response to their comment. This allowed us to connect various quotes to specific pseudonyms, although a few remained anonymous. This study received approval from the Health and Behavioral Science Institutional Review Board (HSBS IRB) at the University of Michigan (IRB #HUM00282928).

### Procedure

Data was collected during March–May 2025. Four focus groups were held during weekdays in the evening time, after participants' work and extracurricular/school related activities. Two focus groups were held on Saturday evenings. Participating families were recruited with the support of community partners serving tens of thousands of Latino/a families who distributed digital flyers with the study information within their networks. Our research team also recruited through social media, advertising our interest in interviewing Latino/a parents and their adolescents ages of 14 and 17 to learn about their perspectives and experiences about mental health and access to care. Interested individuals contacted the study team via text or phone call. Our team utilized a google voice number so that all bilingual team members could answer calls and texts. Once our study team was contacted, we assessed participants' eligibility through a brief screening protocol via phone call with the parents. During the screening, parents were asked if they and their adolescent identified as Latino/a, and their adolescent's age. During this initial phone call with families, team members instructed the parents to choose a pseudonym for them. Adolescents were also required to choose one. Parental verbal consent and verbal assent for their adolescent participation was acquired during that initial phone call. Adolescent verbal consent was also obtained during the same or in a follow up phone call. Copies of the consent/assent forms were texted to participants. Pseudonyms and phone numbers were the only source of information saved from participants. Upon focus groups completion, parents received $50 and adolescents $40 incentives delivered through a digital gift card sent via text message. At that time, their phone numbers were immediately deleted from our database and from any call or text history on google voice. This measure ensured that the study team did not keep any records that could be traced back to the participants. As another measure of data protection, a waiver of signature in the consent and assent forms was obtained from the IRB. Participants indicated their consent/assent by orally saying yes to the question confirming their agreement to participating in a focus group and audio recording. This study has received funding from The National Institute on Minority Health and Health Disparities (NIMHD) and it is covered by a Certificate of Confidentiality from the National Institutes of Health.

Prior to joining the focus group, participants were instructed to keep their cameras off and switch their screen name to their chosen pseudonyms. Participants were asked to refrain from mentioning any identifying information during the focus groups such as names, schools attended by the children, addresses, or their workplace information. The focus group protocol inquired about parents' and adolescents' knowledge and perception of mental illnesses (e.g., depression, anxiety), medications, individual and cultural negative perceptions of mental health as well as access to mental health services. We asked questions such as: “If you ever experience mental health struggles, do you know what to do to get help?” and “How do you feel about talking to your parents about mental health?”. The audio was transcribed and translated by a professional company that provided a nondisclosure agreement to their clients. The first author of the study, who is also a Latina immigrant with experience conducting qualitative research, conducted all the focus groups. She kept her camera on in an effort to establish rapport with participants. Two bilingual research assistants participated in all focus groups taking notes and keeping track of the chat. These field notes were used to support the analysis.

### Data management and analysis

**FIGURE 1:**
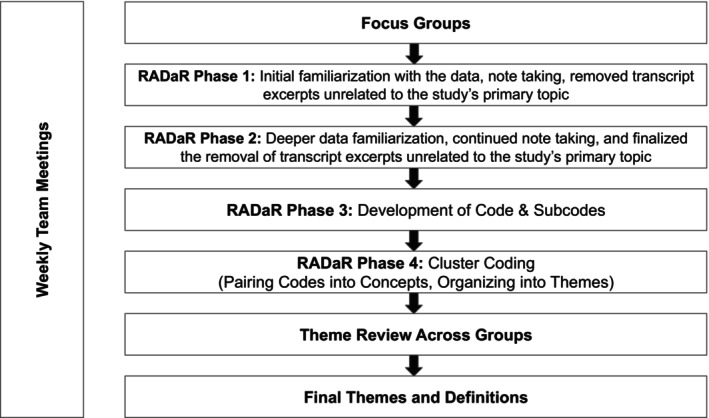
Coding and theme development. This figure illustrates the analytic process used to develop final themes from focus group transcripts. Using a phenomenological approach, the analysis team applied the Rapid and Accelerated Data Reduction (RADaR) technique across four phases to systematically reduce data, develop codes and subcodes, generate cluster themes, and ultimately final themes. This process aligned with Braun and Clarke's six steps of thematic analysis. Coding was conducted in pairs with consensus required at each stage, supported by ongoing team discussion, reflexive review, and consultation with community partners.

We explored individuals' lived experiences with and perceptions of mental health as well as their obstacles and facilitators to seek support. The thematic analysis of interview data was conducted by coding and analyzing the final English focus group transcriptions. The analysis team was composed of five individuals (four were bilingual and three were bicultural) who relied on the Rapid and Accelerated Data Reduction (RADaR) technique for data management (Watkins, 2017), which streamlined the analysis process through organizing the interviews in tables to facilitate revisions (see Figure 1). Through RADaR, the data underwent four phases of analysis. At each phase, the analysis team is paired to check in with their partner and does not move on to the next phase of RADaR until they reach an agreement. This process followed Braun & Clark's six steps of thematic analysis (2006).

During the first phase, the analysis team read the transcripts, took notes throughout, and worked in pairs to confirm excerpts from the transcripts that did not directly relate to the study's primary topic which were removed from the analysis files. Phase two underwent the same process of notetaking, and identifying and removing data that did not pertain to the research question upon team agreement. These first two phases follow the first step of thematic analysis outlined by Braun and Clark (2006). During phase three of RADaR, the analysis team developed a database of codes and subcodes based on their notes and familiarity with the data. Codes and subcodes were then assigned to each line of data drawing from the database, which included codes, subcodes, and their definitions, exemplifying Braun & Clark's second step of thematic analysis (2006).

The next phase of RADaR followed the third step of thematic analysis which consisted of cluster coding, the methodic pairing of codes into concepts and the subsequent organization of cluster themes. In the fourth step of thematic analysis, the themes were reviewed and each cluster theme was supported by concepts and quotes from the raw transcript of focus group sessions, as they are intended to capture broad but specific and distinct themes which are derived from the original transcript data. This process was repeated for each of the six focus groups, then we paired the two parent, two adolescent, and two parent‐adolescent focus groups to identify final themes for each pairing. Final themes with definitions were generated by the data analysis team cross‐referencing cluster themes across all six focus groups, which followed the fifth step of thematic analysis (Braun & Clark, 2006; Bhattacharya, 2017). The sixth and final step of thematic analysis consisted of developing a document with the final themes, how they fit together, and an explanation of each one of them, which provided a clear picture of the entire analysis. This document also included exemplary quotes for each of the themes. The analysis was an iterative process with ongoing discussion and revision from team members (Braun & Clark, 2006). See Table 2 for examples of some initial codes that led to our final themes.

**TABLE 2:** Examples of codes leading to themes, with a representative quote.

Codes	Themes	Representative quote
*Barriers*: Recognizing barriers in addressing or discussing mental health challenges	Practical barriers to mental health help‐seeking	Especially as Hispanic people here in the United States and we don't have access to a lot of information, the language, so to begin with, if we have little bit of time and we're just arriving, then to find the right therapist. To try with one after one, therapists are different here as professionals, unless like in Mexico that is where I have this knowledge from, so they're not the same. (Parent)
*Mental health literacy*: Parent/Adolescents demonstrates mental health literacy or identifies lack of literacy as a barrier to discussing and addressing mental health issues	Mental health literacy and negative perceptions of mental health as barriers to help‐seeking	Yes, I think that it also should be the part of a teen boy that also teaches the part of how the world see us, that a man shouldn't be depressed or sad, that we should only be manly. (Anonymous)
*Stigma*: Parents/Adolescents acknowledge how stigma impacts a person, including their willingness to disclose mental health challenges, seek treatment, or have conversations about it.		
*Facilitators*: Recognizing facilitators in addressing or discussing mental health challenges	Supportive parenting, trust, and communication as key facilitators of support‐seeking	I think that a strong and supportive community is one of the most important things for teens with depression or anxiety. (Adolescent)
Healing: Teen shares about their conceptualization of mental health healing		
*Health service concerns*: Parents and teens have knowledge gaps and share concerns regarding therapy, medication, or other services to address mental health challenges	Navigating medication and treatment decisions	If they open up and tell their parents or parents notice that they have mental health symptoms, maybe one of the difficulties would be not having medical insurance or not having money to pay for therapy or things that could help them. (Parent)

RADaR requires that team members check in with one another at every step of the coding process which ensures strong interrater reliability. They keep track of areas of congruence and disagreements as well as how the disagreements are resolved before moving to the next phase of the analysis. When faced with disagreements, team members refer to their notes, the codebook, and the original interview passage, followed by a discussion of their interpretation of participants' responses. These discussions were typically completed in weekly 60‐min meetings. At times more time was needed, particularly during Phases 3 and 4. This collaborative reflexivity process with extensive discussion and clear communication between team members ensures rigor in the analysis (Olmos‐Vega et al., 2023). In addition, the results were shared with our community partners who provided us with important feedback that enhanced the study's validity.

## RESULTS

The findings from the focus groups provide insights into the complex barriers and facilitators which influence discussions about mental health and support‐seeking behaviors among Latino/a adolescents and parents. Thematic analysis revealed four main themes across the focus groups: (1) Practical barriers to mental health support; (2) Mental health literacy and negative perceptions of mental health as barriers to help‐seeking; (3) Supportive parenting and trust; and (4) Navigating treatment decision‐making (see Table 3).

**TABLE 3:** Core themes, with phenomenological definition, and representative quote.

Themes	Phenomenological definition	Representative quote
Practical barriers to mental health help‐seeking	Parents and adolescents experienced help‐seeking as a process filled with obstacles that made mental health support feel unsafe, overwhelming, and out of reach. Logistical barriers such as language limitations, immigration concerns, cost, time, transportation, and limited provider availability were not experienced in isolation, but collectively produced feelings of fear, misunderstanding, and exclusion. As a result, support was perceived as something that existed in theory but was not truly accessible in practice	My sister told me herself—she was trying to buy a medication that she needed. She was having the worst drawbacks from not taking it after a while. When she tried to buy it without her insurance, it was so much more than she buys it with insurance. It's also so much worse now that Trump's trying to take away healthcare providers and insurances like Medicaid, Medicare. There's just so much that's being messed up. (Adolescent)
Mental health literacy and negative perceptions of mental health as barriers to help‐seeking	Parents and adolescents experienced these obstacles as social pressures that made mental health care feel unwelcoming and difficult to approach. Cultural and language differences contributed to feelings of not being understood, while limited knowledge about mental health made it harder to recognize when support was needed. Judgmental attitudes and restrictive gender norms intensified fear of criticism or shame, discouraging adolescents from seeking help	What did I do? I mean, I blamed myself because I said, how is it possible that my son is reaching this point in which he has to be medicated? But time has passed and as I tell you personally, I take antidepressants, anxiolytics. (Parent)
Supportive parenting, trust, and communication as key facilitators of support‐seeking	Parents and adolescents described communication grounded in trust, emotional attunement, and open dialogue as a key facilitator of mental health support‐seeking. High levels of trust fostered feelings of normalization, safety, and self‐advocacy, making adolescents more willing to disclose concerns and seek help. In contrast, low trust was experienced as a barrier that discouraged openness and reduced the likelihood of pursuing support	Yes, I think mainly that knowing that as a parent you are there for them, listening to them, I think that sometimes as a teenager, right now it's difficult for them to open up with their parents. Many can see them as friends, but there is no such relationship, but I think that the key point is to open that communication channel between the father and the son to know that you are there, maybe you don't have the answers, but having that support, knowing that you are there for them, trying to listen to them and maybe not trying to solve their problems, I think that's a key point in terms of communication with the children. (Parent)
Navigating medication and treatment decisions	Parents and adolescents described the medical model as a common framework for understanding mental health and engaging with professional care. While clinical services were viewed as an important option for treatment and recovery, participants expressed concerns about medication related to limited knowledge, perceived artificiality, and potential physical dependence. Families described different levels of care they were willing to pursue, often prioritizing talk therapy or alternative perspectives before medication use	Honestly, at first, when I was told that I should be on medication and that it was suggested to me to be on anti‐depressants, I was really against it. I was actually scared at first ‘cause I didn't like the thought of a pill changing who I was as a person, I guess, in that aspect. Because I feel like medication would just turn me into someone I wasn't because it's changing stuff in my brain. It's getting me more of this chemical or it's getting more of this. That's really not the case; I figured out over time. At first, I was definitely really against it due to the lack of knowledge I had of it. A lot of the just fears of medication I had of not knowing. I think a good thing would be research too as well about those medications. (Adolescent)

### Theme 1: Practical barriers to mental health help‐seeking

Parents and adolescents alike acknowledged the importance of seeking professional mental health support; however, they also identified several logistical and practical barriers that limit access to services, such as immigration‐related risk factors and developmental vulnerabilities.

Logistical barriers highlighted encompassed language, provider availability, health insurance access, costs, and availability of transportation or time. Both parents and adolescents expressed that language barriers serve as a limitation to accessing mental health support, necessitating multilingual providers which are culturally responsive, acknowledging that very few of these providers are present in their communities. Sofia (Parent Group) explained that “the American culture does not know absolutely nothing about our culture, so I tried to take therapy here with a bilingual therapist, but I definitely could not connect.” Additionally, more structural logistical barriers related to affordability of care are linked to health insurance availability, with a lack of health insurance serving as a barrier to receiving healthcare. As Manuela (Parent Group) explained:

Many of the insurance that we have do not cover many exams. … I do not know if it will be due to the fact of the insurance or it will be due to the clinics or health departments that do not accept the insurance.

Adriana (Parent Focus Group) also shared difficulties related to healthcare costs, “The hardest one for most people that don't have a privileged financial situation is, how much is it going to cost to take my child? I can feed him, or I can send him to therapy.”

Furthermore, other aspects of socioeconomic status made it more challenging to access necessary mental health services. Peter (Adolescent Group) noted that “therapy and other mental health support is very expensive for anyone, really,” while Maya (Adolescent Focus Group) described how “time is a restraint on some people. … I never really had time to go to therapy or do stuff like that.” Carolina (Parent Group) also explained that:

There is a waitlist or a very long wait time, like a year‐long when they can begin seeing a child to give them an appointment. Like the person before me said, time is of the essence, the child needs help immediately and the wait they have in the United States to see a psychologist or psychiatrist, it's too much time. That's a barrier.

Such comments illustrate the multifaceted nature of the challenges in seeking and sustaining mental health support.

Andriana (Parent Group) described their daughter's experiences seeking mental health support in their school district:

I noticed she had certain situations, a panic attack at school. … She is being seen by someone and now she has a different situation. But the barriers that I felt were a bit structural here, you think, “Okay, the school is going to help, that's easy.” They do help but they have their limits. It's like, “Yes, come and talk but I can't help you anymore.” You think, “Okay, but you're a certified psychologist and there is a psychology department at school.” “Yes, but I can't help in that.” So, you go to the next one and the next one, and the next one, those are the difficulties of having a situation with mental health here specifically, in this country.

She highlighted this disparity in resource availability (e.g., school counseling resources, community mental health centers, local therapy services, virtual supports), expressing that when it is so challenging to receive services for mental health concerns, they are less likely to engage emotional energy, motivation, and time into help‐seeking. Both parents' and adolescents' frustration and mistrust toward being able to overcome these logistical and practical barriers, leads to their family systems turning away from potential support.

In addition, both adolescents and parents described some of the ways in which immigration serves as an all‐encompassing factor in mental health help‐seeking and service utilization, with practical barriers being far greater for individuals who have immigrated to the United States. Participants identify immigration status as both a risk factor and a social determinant of help‐seeking behaviors, as resources are less accessible and do not always include trustworthy and culturally responsive practices. Keiko (Adolescent Group) reflected that “with Trump being elected and all that and he's against Latinos and Latinas as well … it affected a lot in the past year,” highlighting how sociopolitical factors intersect with immigration experiences to exacerbate community stress and limit perceived access to care. Finally, developmental vulnerabilities, such as peer pressure, bullying, substance use, and the influence of social media and technology were also discussed as having compounding risk for mental health, as exemplified by Rosa (Parent from Parent‐Adolescent Group): “They are exposed to bullying a lot in schools and out in the street, as well as on social media. Depression is something commonly seen in teenagers nowadays.”

Taken together, these quotes illustrate the multifaceted challenges of seeking mental health support. Latino/a parents and adolescents alike acknowledged the importance of professional help, but identified logistical and practical barriers that limit access, including language and provider availability, health insurance access and costs, and limited transportation or time, alongside long waitlists and structural limits within school‐based supports. Participants also described immigration status as both a risk factor and a social determinant of help‐seeking behaviors, with sociopolitical factors exacerbating stress and limiting perceived access to care. Finally, developmental vulnerabilities (e.g., peer pressure, bullying, substance use, and the influence of social media and technology) were discussed as compounding risks for mental health.

### Theme 2: Mental health literacy and negative perceptions of mental health as barriers to help‐seeking

Parents and adolescents describe multiple obstacles that hinder engagement with professional mental health care, including cultural differences, limited knowledge, judgmental attitudes, and restrictive gender norms. Participants discuss the ways in which negative perceptions and bias influence behaviors which may serve as barriers to adolescents seeking support for depression and anxiety, such as communicating with negative judgment, low mental health literacy, or not having access to information, as expressed by one adolescent (Anonymous, Adolescent Group):

I personally think that there is a huge stigma and a sense of shame when you are diagnosed with depression or anxiety in a family that is Latino or Hispanic that isn't accepting at least. Because for my experience at least, it felt like my whole family thought I was crazy for just having those mental illnesses and stuff like that. Or I've even heard other people's experience where their whole family just thinks they're crazy or not okay in the head completely and that they might be dangerous and stuff like that.

Maria (Adolescent Group) elaborated on these negative perceptions, and lack of mental health literacy in parent–child interactions, sharing:

There's a lot of Hispanic parents that are like, “Oh. That doesn't exist or anything. You'll be fine. You'll be getting through it.” I think it's very important to discuss that topic cause I don't think many Hispanics talk about it.

The adolescents believed that negative perceptions of mental health in the community were a result of low mental health literacy. Marie (Adolescent Group) shared:

I think there is stigma in the Latino community because a lot of Latinos aren't

Informed … I learned in school in health class what all these things are, but I feel like

Latinos aren't informed. They don't believe these are mental illnesses but more of emotions or feelings so they don't quite grasp how serious it can really be.

Furthermore, adolescents shared that this minimization of symptoms or mental health concerns led to a reduction in conversation around mental health, instead opting to share with peers, while expressing a desire for these conversations to be had in the household and beyond. As one adolescent (Anonymous, Adolescent Group) explained:

I'd feel better talking to my friends about a mental health condition because—or just people my age in general—because I feel like the majority of the people that are young adolescents have had their fair share of experiences with their own mental health conditions. It's become pretty normalized in young people. I feel like everybody who is of a young age can all at least have a consensus and be there to all help each other because everybody's gone through similar things.

When adolescents were among individuals who normalize or regularly discuss mental health concerns, they were inclined to share their experiences and seek support (either practically or emotionally). Adolescents emphasized that among individuals who have greater mental health literacy, they notice this normalization occurs. The adolescents also discussed how they could identify depression and anxiety symptoms as well as ways to get support. One adolescent (Anonymous, Adolescent Focus Group) explained:

I know that you can seek a therapist, a school counselor, if that's available. There's hotlines. I've heard from my sister—once she was having trouble, and she called a suicide hotline. They had to put her on a waitlist, so sometimes it can be a little harder to get help.

Conversely, parents explored ways in which their difficulty identifying signs and symptoms of depression and anxiety served as a barrier to their child receiving support. A parent (Anonymous, Parent‐Adolescent Group) described that even if their adolescent expressed a concern, they struggle to support them in a developmentally appropriate manner:

The teenager can ask for help but, sometimes as parents, we don't know how to do it. I mean, it's hard to put ourselves in the teenager's shoes, and we expect them to react the same way an adult would, but they don't. For them, it feels like their world is falling apart, and it's not the same. They're not feeling what the parent is feeling. So I think there's a lack of information for parents and how to come down to their level and help them as teenagers when they feel like their world is falling apart.

Further, they explained the ways in which social and parental pressure served a compounding impact and might lead adolescents to feel increased pressure for performance which exacerbates their depressive and anxious symptoms. Manuela (Parent Group) stated:

The fear, the confidence in themselves, the pressure, the demands, as [someone else] said before, that you demand as a mother, to want to give them the opportunity to be better … to overcome, but also to be autonomous, to let them know not to be afraid. And sometimes as mothers, or as parents, it's easier to go too far, to demand a little more, and I feel that in the end we're damaging that relationship … because of the demands … that they be better, but as good Latinos, we do it with love and the conviction that they are better people in the future.

Sofia (Parent Group) expressed guilt at not being able to identify her son's mental health needs and symptoms earlier on in his journey:

What did I do? I mean, I blamed myself because I said, “How is it possible that my son is reaching this point in which he has to be medicated?” But time has passed and as I tell you personally, I take antidepressants, anxiolytics.

Sofia discussed how over time she also began to take prescription medication to manage her mental health. While she had felt guilt for not noticing that her son needed mental health support before requiring medication, by going through that experience they have now come to normalize symptom management strategies. Increased mental health literacy or exposure to treatment opportunities promoted their own help‐seeking and service utilization.

Overall, parents and adolescents described barriers to engaging in professional mental health care, including cultural differences, limited knowledge, and judgmental attitudes. Negative perceptions and low mental health literacy reduced parent‐adolescent conversations about depression and anxiety, often shifting support‐seeking toward peers, while parents reported difficulty identifying symptoms and responding in developmentally appropriate ways. Increased mental health literacy and exposure to treatment opportunities supported greater normalization of symptom management and openness to help‐seeking.

### Theme 3: Supportive parenting, trust, and communication as key facilitators of support‐seeking

Parent and child communication grounded in trust, emotional attunement, and open dialogue emerged as a key facilitator of support‐seeking, fostering normalization, and self‐advocacy. Adolescents and parents alike highlighted levels of trust as being predictive of help‐seeking, with high levels of trust being associated with an elevated likelihood of help‐seeking. Dania (Parent Group) exemplified this, saying:

Knowing that the parents are always there, and they can tell them whatever they want, express themselves. The parents are there, not to judge them but to listen and understand and not have a barrier between a parent and a child that as Latinos we can sometimes be harsher when talking. We need to break that barrier of fear of your parents and have more trust and respect, more than fear.

Adolescents highlighted a disclosure process which is influenced by their familial and parental relationships. Adolescents with past negative and unsupportive parental reactions (e.g., minimization, dismissal, fear of judgment, or misplaced blame) appeared less likely to disclose mental health challenges, whereas adolescents with more positive, supportive, and validating experiences with parents appeared more willing to disclose. Andre (Adolescent Group) described his fears in disclosure:

Maybe they'll blame me on something. Maybe you'll talk about it and they think it's a friend influencing you. They then make you hang out with that friend less or maybe they blame it on your playing video games or being on your phone, then they punish you. … Then you don't wanna talk to them ‘cause you feel like it's gonna be worse’ cause you gonna get punished instead of being helped.

Victoria (Adolescent Group) shared that she found support among her friends for mental health concerns, explaining that disclosing her mental health needs to her parents could change relational dynamics or lead to negative consequences:

I would talk to my friends in one of those situations 'cause … it can be hard to talk about that stuff with your parents, especially because so much depends on them. For me, they're one of the people that I spend the most time with, so if you come out with them with a confession like that about your mental health. It could completely change the way they see you, or your privileges, and how they treat you.

Parents were also able to identify that their adolescent children may be reluctant to disclose their mental health concerns. Parents demonstrated knowledge on how to be supportive to adolescents, including: nonjudgemental listening, open communication, quality time, empathy, encouragement, and being attuned to adolescent's wellbeing. Dimas (Parent Group) described the importance of these relational qualities, stating:

I think mainly that knowing that as a parent you are there for them, listening to them, I think that sometimes as a teenager, right now it's difficult for them to open up with their parents. Many can see them as friends, but there is no such relationship, but I think that the key point is to open that communication channel between the father and the son to know that you are there, maybe you don't have the answers, but having that support, knowing that you are there for them, trying to listen to them and maybe not trying to solve their problems, I think that's a key point in terms of communication with the children.

Parents expressed that even if they don't have all the answers, having supportive qualities promotes conversation with adolescents about their mental health and facilitates help‐seeking behaviors. Furthermore, parents highlighted Latino/a culture as a contextual factor in help‐seeking behaviors, expressing that there are strengths which promote connection and trust. One parent (Anonymous, Parent‐Adolescent Group) shared:

When you go to a hospital, the Latinos go with their loved ones. I've seen people from here who are alone. You see Latino people, and there are four or five people there. That is excellent. It's a very beautiful culture that we have because we support each other, not only as relatives, but as friends, as well. That is something good to point out, always.

Participants highlighted that these cultural factors of compadres and family support serve as facilitators, promoting trust, loyalty, and support. They emphasized the importance of mental health care which extends these types of support into clinical spaces.

Overall, parent–child communication grounded in trust, emotional attunement, and open dialogue emerged as a key facilitator of support‐seeking. Adolescents' disclosure was shaped by prior parental reactions, minimization, dismissal, fear of judgment, or misplaced blame; reduced disclosure and shifted support toward friends, whereas validation and nonjudgmental listening increased willingness to talk and seek help. Parents also emphasized being available, keeping open communication, and drawing on Latino/a strong family support to break barriers of fear and extend trust.

### Theme 4: Navigating medication and treatment decisions

Parents and adolescents highlighted the role of the medical model (terminology and framework) in understanding and addressing mental health, viewing professional care as a key avenue for treatment engagement/adherence, rehabilitation, and recovery. Participants raised concerns for medication, including lack of literacy around medication use, concerns for the composition of medication (being “unnatural” or “chemical”), and concerns for physiological dependence on medication. They also considered complementary perspectives, including spiritual frameworks and personal or observed experiences, while expressing caution regarding the use of medication, as expressed by Peter (Adolescent Group):

For me, I feel that it's a really big risk trying to take anti‐depressants. I think one big step to deciding whether I would be heavily educating myself on the side effects and all of that come with the anti‐depressants.

Similarly, Adriana (Parent Group) reflected on her decision‐making process when presented with the possibility of medicating her adolescent child:

I would say, ‘Okay, if this is absolutely necessary, yes,’ because it changes the chemistry in the brain and it can create a dependency or when you can't find the medication, you get that withdrawal and that is worse. When someone doesn't take an antidepressant, they go through psychosis, they go through a state of anxiety that is much bigger…yes, if it's absolutely necessary. Any other way, okay, he needs it now but tell me how long and he can leave it.

While Sofia (Parent Group) described her reaction upon learning her son needed medication:

And when the psychiatrist of my son and the therapist, between the two of them, communicated and had shared that they were going to start giving medication to my son, that day I left therapy and I cried, I cried bitterly thinking how it is possible that they are going to medicate my son, and I told my husband and one of my sisters and I told them and I cried, I cried a lot because I said, ‘What did I fail as a mother? What am I doing?’

Participants also described their individual hierarchies or levels of care that they seek (e.g., talk therapy, medication, partial hospitalization, inpatient), such as prioritizing talk therapy over medication use. Many adolescent participants expressed feeling an initial resistance to medication, citing fear and limited mental health literacy on psychopharmacological intervention. One adolescent (Anonymous, Adolescent Group) reflected on this early reluctance, sharing:

Honestly, at first, when I was told that I should be on medication and that it was suggested to me to be on anti‐depressants, I was really against it. I was actually scared at first cause I didn't like the thought of a pill changing who I was as a person… I feel like medication would just turn me into someone I wasn't because it's changing stuff in my brain… At first, I was definitely really against it due to the lack of knowledge I had of it.

The participant further described how increased access to information and education shifted their perspective: “That's really not the case, I figured out over time… I think a good thing would be research too as well about those medications.”

Luis (Adolescent Group) explained how they got to the point of accepting the use of medication:

For me, one of the reasons that really got me on medication was after being recommended a couple of times—like three, four times—and me saying, ‘No, I wanna try my best to do it naturally. I think I got this. I think I got this.’ Constantly having to repeat that conversation, constantly being told, ‘Hey. You need medication,’ and constantly realizing that being alone and not being on that is not helping and that I'm making zero progress is honestly what helped me push forward and take that medication.

These reflections show an evolution in acceptance of treatment and medication. While participants may not have all the information, they were open to the possibility and described a gradual introduction to mental health treatment options, as Marie (Adolescent Group) expressed:

Personally, I don't think it goes away on its own. I think you do need treatment, not necessarily medication, but a change in lifestyle that won't expose you as much to the situations that may put people—that make them feel like they have to feel anxious or depressed or overwhelmed with negative feelings and stuff.

The participants emphasized agency, independence, and action (rather than passivity) as playing a role in their ability to seek and utilize treatment services. Furthermore, they described a hierarchical pattern for help‐seeking behavior, prioritizing self‐management and nonpharmacological interventions prior to medication management options. Participants demonstrated an ongoing negotiation between medical and interpersonal frameworks for managing treatment.

## DISCUSSION

This study sought to determine facilitators and barriers for Latino/a adolescent help‐seeking behaviors, particularly with adolescents discussing their mental health needs with their parents and use of mental health services. Disclosure is an important aspect of adolescents receiving mental health care; however, there is limited research on what impacts disclosure and engagement with mental health care among Latino/a adolescents (Lu et al., 2021).

Through our analysis, four key themes were highlighted. Theme 1 (*Practical Barriers to Mental Health Help‐Seeking*) refers to the mutual acknowledgement among adolescents and parents about seeking professional mental health support and the key logistical and practical barriers they face. Theme 2 (*Mental Health Literacy and Negative Perceptions of Mental Health as Barriers to Help‐Seeking*) focuses on obstacles that limit engagement with professional mental health care including judgment, cultural and language differences, and limited mental health literacy. Theme 3 (*Supportive Parenting, Trust, and Communication as Key Facilitators of Support‐Seeking*) illustrates how support‐seeking, fostering normalization, and self‐advocacy can be facilitated if they are grounded in communication that is anchored in trust, emotional attunement, and open dialogue. Lastly, Theme 4 (*Navigating Medication and Treatment Decisions*) where participants describe professional mental health support as a viable option for treatment and highlight how the levels of care that they need can be influenced by potential concerns, and therefore prefer one form of treatment over another (e.g., talk therapy over medication).

While synthesized as four separate themes, the combination of these themes illustrates a fuller picture on the facilitators and barriers for Latino/a parent–adolescent mental health discussions and help‐seeking. Participants revealed that while adolescents and parents both understand the components of a successful parent‐adolescent combination, the barriers they encounter make it difficult to seek mental health treatment. Further, adolescents' perception of available care was affected by these logistical and practical barriers, especially when they witnessed family or other close community members face such challenges. This suggests that the family's frustrations in encountering these logistical and structural barriers may lead to their family turning away from potential support.

Participants reported various barriers when attempting to seek mental health support. The shared understanding between adolescents and parents alike is that these barriers are structural, which makes it difficult to engage with services. While some of these factors are unique to the Latino/a community (e.g., cultural, linguistic barriers, and contemporary political issues), some of these other barriers are not. Research has shown that some of the most prevalent barriers to access healthcare as a whole are issues with affordability, including social and environmental factors (Coombs et al., 2021). Specifically, to underserved and disenfranchised communities, such as the Latino/a community, our findings are in alignment with previous studies that have also highlighted lack of insurance, lack of community‐based interventions, and stigma as barriers for accessing mental health services. This also includes mental health workforce shortages and geographical maldistribution of providers as barriers (Mongelli et al., 2020).

There was a strong emphasis on the role that limited mental health literacy can play in help‐seeking. This expressed itself in the form of negative perceptions about mental health, fear of judgment, cultural and language differences, as well as limited knowledge on accessing support. The ways that negative perceptions about mental health may influence behaviors have been cited in other studies. One study highlighted that more than 70% of people with mental illness receive no treatment with evidence suggesting that this is due to (1) lack of knowledge on symptoms, (2) ignorance in how to access treatment, (3) prejudice against people who have mental illness, and (4) expectation of discrimination against those with a diagnosis (Henderson et al., 2013). However, a systematic review suggested that negative perceptions about mental health can have a small to moderate negative impact on help‐seeking behaviors, ranking it as the fourth out of 10 barriers (Clement et al., 2015). Coupled with our findings, it appears that negative perceptions of mental health are an important barrier in seeking support but may not be as limiting as mental health literacy. Unfortunately, these factors oftentimes co‐exist and thus their barriers are compounded.

Participating adolescents appeared to perceive that they had, in part, sufficient information to understand the symptoms and severity of depression and anxiety, even suggesting they may have more knowledge about mental health than their parents. Adolescents were generally able to describe anxious and depressive symptoms, as well as identify sources of support, such as crisis hotlines, school counselors, and mental health therapists. This, along with low levels of internalized negative mental health perceptions, led to participating adolescents emphasizing relational quality and anticipated outcome being the primary factors in their decision‐making process. Indeed, supportive parenting–based on trust, empathy, nonjudgmental listening, and validation–was a significant component to whether they would disclose mental health challenges with their parents. On the other hand, when adolescents anticipated negative responses by their parents, including minimization or dismissal, adolescents were less likely to disclose. Adolescents' assessment of the relationship quality and the anticipated outcome with their caregivers appeared to be heavily weighed in this context. This is consistent with the DD‐MM which indicates that after considering the health information, the person disclosing assesses the receiver, the individual with whom the information will be shared, and examines the relational quality, anticipated response, and anticipated outcome (Greene, 2009). Notably, parents also reported that supportive parenting strategies were helpful in facilitating dialogue with their adolescent children.

Participants noted that familismo also plays a role in both facilitating and hindering mental health help‐seeking outcomes. Participants emphasized their desire for privacy and autonomy in sharing their mental health concerns with others, naming that familismo or a more collective family reputation makes this sometimes harder to share. Despite this, participants (particularly parents) also emphasized how this is a benefit, exemplifying this through vibrant community support for individuals who are in distress. This interplay sometimes complicates adolescents' disclosure of mental health concerns.

Negative perceptions about mental health by others, specifically their parents, affected adolescents' decision to disclose. Adolescents believed that parents' minimization of mental health or negative perceptions of mental health are due to low mental health literacy. Interestingly, parents also recognized how negative perceptions of mental health and poor mental health literacy negatively affected their ability to support their adolescent children. This suggests a need for a mental health literacy education tool that builds upon the individual's initial mental health knowledge while addressing negative perceptions of mental health. This, coupled with strategies that help mitigate logistical and structural barriers, can help achieve successful engagement with mental health services among Latino/a adolescents.

By gathering information from both parents and adolescents, we were able to unpack the parent–child dynamics that impact the family's mental health discussions and engagement in help‐seeking behaviors. This intergenerational approach incorporated both parents' and adolescents' perspectives, which appears lacking in literature examining Latino/a families in particular. To our knowledge, the DD‐MM had not previously been used with Latino/a adolescents, and though our results appear consistent with the model, there are aspects of disclosure that are not captured in the DD‐MM (Greene, 2009). This supports a previous study's recommendation that a variation of the model could be called the Adolescent's Mental Health Disclosure Decision‐Making Model (Rasmussen et al., 2022). In addition to the aforementioned results, our study further indicates low overall communication about mental health among parents and adolescents, thus highlighting the need for more studies focusing on this topic. Additionally, despite adolescents reporting having greater mental health literacy than their parents, they were also hesitant to use medication; however, our results also suggest that with accessible and culturally responsive education, Latino/a adolescents and parents are likely to be more willing to take medication when it is mutually agreed that it is medically necessary (Wisdom et al., 2006).

## IMPLICATIONS FOR PRACTITIONERS

Our results indicate that Latino/a adolescents experiencing depression and/or anxiety would benefit from having culturally responsive interventions at first contact with a doctor or primary care setting, including screening, psychoeducation, and ongoing referrals. Moreover, the parent–child relational dynamic was an assessment made by adolescents that significantly influenced whether they would consider disclosing their mental health challenges. Parents, on the other hand, also recognized they might not have the tools to adequately support their adolescents through mental health challenges. Though there are few interventions for parent–child relationships, this suggests families would greatly benefit from a holistic parent–child relational intervention that is culturally responsive and that supports family communication about mental health while addressing mental health literacy and negative perceptions of mental health.

Another implication is the potential use of a facilitator, such as a Community Health Worker (CHW), which may be helpful to support parent‐adolescent conversations about mental health. Previous studies have shown that CHW's can play a key role in mental health conversations due to their cultural understanding, personal ties to their respective communities, and the link they serve between communities and health care (Knowles et al., 2023). Within the Latino/a population, a different systematic literature review found that the majority of 27 studies, 25 of which were trials, whose interventions were delivered by CHW's, led to significant improvement of mental health symptoms (Gustafson et al., 2025). This suggests that in the context of a holistic parent–child intervention that requires cultural responsiveness and supports family community, CHW's could play a key role.

## LIMITATIONS

Limitations in this study may stem from five different areas. The first is the geographic location of where participants were recruited from. The Latino/a population is spread across the entire US. Considering this, the research team recruited participants from Michigan and Texas. While these are two different regions of the United States, it fails to capture the full scope of the Latino/a population in the US, limiting the generalizability to Latino/a populations living in other parts of the country. For example, our findings may not generalize to Cubans or Puerto Ricans given that they are less likely to reside in these states. Secondly, across all participants, 65% were of Mexican origin, and across parents, 87% were mothers. This limits the generalizability to other groups. Additionally, with the small number of fathers and father figures, our findings largely capture a maternal perspective, which in turn does not encompass the entire parental perspective.

Thirdly, in an attempt to make participation as accessible as possible, focus groups were conducted in a virtual format. While this may address contemporary political issues that prevent individuals from the Latino/a community from feeling safe participating in person, it comes with certain shortcomings. This includes the difficulty of building trust between the interviewer and participants, creating a space for organic group interaction and conversations to happen, as well as creating impartiality between participants that have the capacity to join in a virtual format versus those that would prefer an in‐person format. Fourthly, two of the focus groups contained both parents and adolescents that came from the same family. This may have influenced what parents and adolescents in particular felt comfortable disclosing due to fear of repercussions. To address this limitation, separate adolescent and parent focus groups were added to provide more opportunities to freely share. Finally, the participating parents and adolescents were not evaluated for the extent of mental health literacy, and recruitment was community‐based and thus may have captured a sample which had higher mental health literacy due to willingness and desire to share experiences related to mental health. To address this limitation, future studies may consider evaluating for and diversifying samples to reflect variance in mental health literacy.

Future research should seek how initial themes present across different regions of the United States, and how facilitators between parent‐adolescent conversations evolve with time. It should also seek to provide culturally relevant tools to decrease barriers and increase mental health literacy to increase access to the care that Latino/a adolescents may need. Potential ways of achieving this could include developing an entertainment‐education intervention (E–E) such as a fotonovela that is culturally relevant and delivered by a facilitator such as a CHW. In the past, fotonovelas have been utilized as an educational tool for the Latino/a community (Valle et al., 2006), with research supporting that they could be an effective tool to address these needs (Cabassa et al., 2012). In connection with this, CHWs have been known to be a key bridge between Latino/a communities and health, and support the increase of preventative care (Knowles et al., 2023). Therefore, an E–E intervention that is culturally relevant to Latino/a and implemented with the support of facilitators such as CHWs may increase mental health literacy, combat negative perceptions, and encourage adolescents to learn how to disclose mental health concerns with parents.

## CONCLUSION

Latino/a adolescents disproportionately face significant mental health challenges, yet are less likely to seek and access mental healthcare. Drawing from the Disclosure Decision‐Making Model (DD‐MM), we explored the facilitators and barriers of Latino/a adolescents' help‐seeking behaviors, focusing on adolescents discussing their mental health needs with parents and use of mental health services. Results offer insight into the Latino/a experience navigating mental health through four themes: (1) Practical Barriers to Mental Health Help‐Seeking, (2) Mental Health Literacy and Negative Perceptions of Mental Health as Barriers to Help‐Seeking, (3) Supportive Parenting, Trust, and Communication as Key Facilitators of Support‐Seeking, and (4) Navigating Medication and Treatment Decisions. Findings highlight the need for interventions that reduce negative perceptions about mental health, improve mental health literacy, and strengthen parent‐adolescent communication to promote Latino/a adolescents' access to mental health services. This can include developing an entertainment‐education intervention (E–E), such as a fotonovela, which has previously been utilized as an effective tool for the Latino/a community to address mental health needs (Cabassa et al., 2012; Valle et al., 2006).

## AUTHOR CONTRIBUTIONS

**Fernanda Lima Cross:** Conceptualization; investigation; funding acquisition; writing – original draft; methodology; writing – review and editing; formal analysis; project administration; supervision; resources. **Joel Lucio:** Writing – original draft; writing – review and editing; formal analysis; project administration. **Amanda Webster:** Writing – original draft; writing – review and editing; formal analysis. **Zachary Sessa:** Formal analysis; writing – review and editing; writing – original draft. **Irving Suarez:** Writing – original draft; writing – review and editing; formal analysis. **Kenneth Resnicow:** Writing – original draft; writing – review and editing.

## FUNDING INFORMATION

Research reported in this publication was supported by the National Institute on Minority Health and Health Disparities of the National Institutes of Health under Award Number 5K01MD019325. The content is solely the responsibility of the authors and does not necessarily represent the official views of the National Institutes of Health.

## Data Availability

The data that support the findings of this study are available on request from the corresponding author. The data are not publicly available due to privacy or ethical restrictions.

## References

[jora70232-bib-0001] Benuto, L. T. , Gonzalez, F. , Reinosa‐Segovia, F. , & Duckworth, M. (2019). Mental health literacy, stigma, and behavioral health service use: The case of Latino/a and non‐Latino/a whites. Journal of Racial and Ethnic Health Disparities, 6(6), 1122–1130. 10.1007/s40615-019-00614-8 31327136

[jora70232-bib-0002] Bhattacharya, K. (2017). Fundamentals of qualitative research: A practical guide (1st ed.). Routledge. 10.4324/9781315231747

[jora70232-bib-0003] Bismar, D. (2018). Mental illness stigma, parent‐child communication, and help‐seeking of young American adults with immigrant parents. Doctoral diss., https://digital.library.unt.edu/ark:/67531/metadc1248426/m2/1/high_res_d/BISMAR‐THESIS‐2018.pdf

[jora70232-bib-0004] Borrero, E. , & Przeworski, A. (2025). A systematic review of evidence‐based treatments for depression in Latinx youth. Child & Family Behavior Therapy, 47(3), 283–340.

[jora70232-bib-0062] Braun, V. , & Clarke, V. (2006). Using thematic analysis in psychology. Qualitative Research in Psychology, 3(2), 77–101. 10.1191/1478088706qp063oa

[jora70232-bib-0005] Brewer, K. B. , Washburn, M. , Yu, M. , Giraldo‐Santiago, N. , Pickford, M. , Hostos‐Torres, L. R. , & Gearing, R. E. (2024). Stigma toward families with mental health problems in Latino communities. Families in Society: The Journal of Contemporary Social Services, 106(4), 1084–1097. 10.1177/10443894241237018

[jora70232-bib-0006] Cabassa, L. J. , Molina, G. B. , & Baron, M. (2012). Depression fotonovela: Development of a depression literacy tool for Latinos with limited English proficiency. Health Promotion Practice, 13(6), 747–754. 10.1177/1524839910367578 21051325 PMC3071859

[jora70232-bib-0007] Cabassa, L. J. , Zayas, L. H. , & Hansen, M. C. (2006). Latino adults' access to mental health care: A review of epidemiological studies. Administration and Policy in Mental Health, 33(3), 316–330. 10.1007/S10488-006-0040-8 16598658 PMC2551758

[jora70232-bib-0008] Caplan, S. , Paris, M. , Whittemore, R. , Desai, M. , Dixon, J. , Alvidrez, J. , & Scahill, L. (2011). Correlates of religious, supernatural, and psychosocial causal beliefs about depression among Latino immigrants in primary care. Mental Health, Religion and Culture, 14(6), 589–611. 10.1080/13674676.2010.497810

[jora70232-bib-0009] Carpenter, A. M. , & Theiss, J. A. (2023). Modeling the factors associated with topic avoidance about mental health: Depressive symptoms, information and relationship assessments, and efficacy. Southern Communication Journal, 88(1), 16–29. 10.1080/1041794X.2022.2118365

[jora70232-bib-0010] Chaudoir, S. R. , & Fisher, J. D. (2010). The disclosure processes model: Understanding disclosure decision making and postdisclosure outcomes among people living with a concealable stigmatized identity. Psychological Bulletin, 136(2), 236–256. 20192562 10.1037/a0018193 PMC2922991

[jora70232-bib-0011] Chavira, D. A. , Bantados, B. , Rapp, A. , Firpo‐Perretti, Y. M. , Escovar, E. , Dixon, L. , Drahota, A. , & Palinkas, L. (2017). Parent‐reported stigma and child anxiety: A mixed methods research study. Children and Youth Services Review, 76, 237–242. 10.1016/j.childyouth.2017.03.013 29576669 PMC5860669

[jora70232-bib-0012] Clement, S. , Schauman, O. , Graham, T. , Maggioni, F. , Evans‐Lacko, S. , Bezborodovs, N. , Morgan, C. , Rüsch, N. , Brown, J. S. , & Thornicroft, G. (2015). What is the impact of mental health‐related stigma on help‐seeking? A systematic review of quantitative and qualitative studies. Psychological Medicine, 45(1), 11–27. 10.1017/S0033291714000129 24569086

[jora70232-bib-0013] Collado, A. , Zvolensky, M. , Lejuez, C. , & MacPherson, L. (2019). Mental health stigma in depressed Latinos over the course of therapy: Results from a randomized controlled trial. Journal of Clinical Psychology, 75(7), 1179–1187. 10.1002/jclp.22777 30951609 PMC6559847

[jora70232-bib-0014] Coombs, N. C. , Meriwether, W. E. , Caringi, J. , & Newcomer, S. R. (2021). Barriers to healthcare access among U.S. adults with mental health challenges: A population‐based study. SSM ‐ Population Health, 15, 100847. 10.1016/j.ssmph.2021.100847 34179332 PMC8214217

[jora70232-bib-0015] Corrigan, P. W. , Druss, B. G. , & Perlick, D. A. (2014). The impact of mental illness stigma on seeking and participating in mental health care. Psychological Science in the Public Interest, 15(2), 37–70. 10.1177/1529100614531398 26171956

[jora70232-bib-0016] Cortés‐García, L. , Hernández Ortiz, J. , Asim, N. , Penner, F. , Sharp, C. , Sales, M. , & Villareal, R. (2022). COVID‐19 conversations: A qualitative study of majority Hispanic/Latinx youth experiences during early stages of the pandemic. Child & Youth Care Forum, 51(4), 769–793. 10.1007/s10566-021-09653-x 34602804 PMC8477975

[jora70232-bib-0017] DeFreitas, S. C. (2022). Mental health stigma in the Latino/a population: Treatment implications and future directions. In R. Castilla‐Puentes & T. Falcone (Eds.), Mental health for Hispanic communities (pp. 25–41). Springer. 10.1007/978-3-031-13195-0_3

[jora70232-bib-0018] Devi, S. , Joseph, J. , Devi, R. , & Singh, B. (2025). Mental health literacy and treatment compliance among clients with mental illness in India. Journal of Psychiatry Spectrum, 4(3), 271–272. 10.4103/jopsys.jopsys_82_24

[jora70232-bib-0019] Diala, C. , Muntaner, C. , Walrath, C. , Nickerson, K. J. , LaVeist, T. A. , & Leaf, P. J. (2000). Racial differences in attitudes toward professional mental health care and in the use of services. American Journal of Orthopsychiatry, 70(4), 455–464. 10.1037/h0087736 11086524

[jora70232-bib-0020] Dixon De Silva, L. E. , Ponting, C. , Ramos, G. , Guevara, M. V. C. , & Chavira, D. A. (2020). Urban Latino/a parents' attitudes towards mental health: Mental health literacy and service use. Children and Youth Services Review, 109, 104719. 10.1016/j.childyouth.2019.104719 37842164 PMC10575228

[jora70232-bib-0021] Elkington, K. S. , Hackler, D. , McKinnon, K. , Borges, C. , Wright, E. R. , & Wainberg, M. L. (2012). Perceived mental illness stigma among youth in psychiatric outpatient treatment. Journal of Adolescent Research, 27(2), 290–317. 10.1177/0743558411409931 33840885 PMC8031474

[jora70232-bib-0022] Greene, K. (2009). An integrated model of health disclosure decision‐making. In T. D. Afifi & W. A. Afifi (Eds.), Uncertainty, information management, and disclosure decisions: Theories and applications (pp. 226–253). Routledge/Taylor & Francis Group. 10.4324/9780203933046

[jora70232-bib-0023] Greene, K. , Magsamen‐Conrad, K. , Venetis, M. K. , Checton, M. G. , Bagdasarov, Z. , & Banerjee, S. C. (2012). Assessing health diagnosis disclosure decisions in relationships: Testing the disclosure decision‐making model. Health Communication, 27(4), 356–368. 10.1080/10410236.2011.586988 21992531

[jora70232-bib-0024] Gustafson, E. L. , Moses, J. O. , Pimentel, E. , Lakind, D. , Uribe, V. , Thorpe, D. , Bobadilla, G. , Caglianone, L. , Dickinson, G. J. , Smith, D. , Westrick, J. , & Sánchez‐Johnsen, L. (2025). Community health worker‐delivered mental health interventions for Latine populations in the U.S. A systematic literature review. Administration and Policy in Mental Health and Mental Health Services Research, 52(6), 1174–1198. 10.1007/s10488-025-01459-6 40690129 PMC12628377

[jora70232-bib-0025] Henderson, C. , Evans‐Lacko, S. , & Thornicroft, G. (2013). Mental illness stigma, help seeking, and public health programs. American Journal of Public Health, 103(5), 777–780. 10.2105/AJPH.2012.301056 23488489 PMC3698814

[jora70232-bib-0026] Hoffmann, J. A. , Alegría, M. , Alvarez, K. , Anosike, A. , Shah, P. P. , Simon, K. M. , & Lee, L. K. (2022). Disparities in pediatric mental and behavioral health conditions: A state‐of‐the‐art review. Pediatrics, 150(4), e2022058227. 10.1542/peds.2022-058227 36106466 PMC9800023

[jora70232-bib-0027] Interian, A. , Ang, A. , Gara, M. A. , Link, B. G. , Rodriguez, M. A. , & Vega, W. A. (2010). Stigma and depression treatment utilization among Latinos: Utility of four stigma measures. Psychiatric Services, 61(4), 373–379. 10.1176/ps.2010.61.4.373 20360276 PMC3222155

[jora70232-bib-0028] Jorm, A. F. , Korten, A. E. , Jacomb, P. A. , Christensen, H. , Rodgers, B. , & Pollitt, P. (1997). “Mental health literacy”: A survey of the public's ability to recognise mental disorders and their beliefs about the effectiveness of treatment. Medical Journal of Australia, 166(4), 182–186. 10.5694/j.1326-5377.1997.tb140071.x 9066546

[jora70232-bib-0029] Knowles, M. , Crowley, A. P. , Vasan, A. , & Kangovi, S. (2023). Community health worker integration with and effectiveness in health care and public health in the United States. Annual Review of Public Health, 44(1), 363–381. 10.1146/annurev-publhealth-071521-031648 37010928

[jora70232-bib-0031] Lu, W. , Todhunter‐Reid, A. , Mitsdarffer, M. L. , Muñoz‐Laboy, M. , Yoon, A. S. , & Xu, L. (2021). Barriers and facilitators for mental health service use among racial/ethnic minority adolescents: A systematic review of literature. Frontiers in Public Health, 9, 641605. 10.3389/fpubh.2021.641605 33763401 PMC7982679

[jora70232-bib-0032] Marín, G. , & Marín, B. V. (1991). Research with Hispanic populations. Sage Publications.

[jora70232-bib-0063] McCord, A. L. , Draucker, C. B. , & Bigatti, S. (2019). Cultural stressors and depressive symptoms in Latino/a adolescents: An integrative review. Journal of the American Psychiatric Nurses Association, 25(1), 49–65. 10.1177/1078390318778885 29862864 PMC7700804

[jora70232-bib-0033] Mongelli, F. , Georgakopoulos, P. , & Pato, M. T. (2020). Challenges and opportunities to meet the mental health needs of underserved and disenfranchised populations in the United States. Focus: Journal of Life Long Learning in Psychiatry, 18(1), 16–24. 10.1176/appi.focus.20190028 32047393 PMC7011222

[jora70232-bib-0034] Nogueira, A. L. , & Schmidt, I. (2022). “One cannot make it alone”: Experiences of a community faith‐based initiative to support Latino mental health. Social Work in Mental Health, 20(6), 645–671. 10.1080/15332985.2022.2049953

[jora70232-bib-0035] Olmos‐Vega, F. M. , Stalmeijer, R. E. , Varpio, L. , & Kahlke, R. (2023). A practical guide to reflexivity in qualitative research: AMEE guide No. 149. Medical Teacher, 45(3), 241–251. 10.1080/0142159X.2022.2057287 35389310

[jora70232-bib-0036] Pahwa, R. , Fulginiti, A. , Brekke, J. S. , & Rice, E. (2017). Mental illness disclosure decision making. American Journal of Orthopsychiatry, 87(5), 575–584. 10.1037/ort0000250 28394157

[jora70232-bib-0037] Parra‐Cardona, J. R. , & DeAndrea, D. C. (2016). Latinos' access to online and formal mental health support. The Journal of Behavioral Health Services & Research, 43(2), 281–292. 10.1007/s11414-014-9420-0 24938931 PMC4270952

[jora70232-bib-0038] Parsai, M. , Voisine, S. , Marsiglia, F. F. , Kulis, S. , & Nieri, T. (2008). The protective and risk effects of parents and peers on substance use, attitudes, and behaviors of Mexican and Mexican American female and male adolescents. Youth & Society, 40(3), 353–376. 10.1177/0044118X08318117 PMC2686611 19478992

[jora70232-bib-0039] Pérez‐Flores, N. J. , & Cabassa, L. J. (2021). Effectiveness of mental health literacy and stigma interventions for Latino/a adults in the United States: A systematic review. Stigma and Health, 6(4), 430–439. 10.1037/sah0000343 35368243 PMC8974450

[jora70232-bib-0040] Piña, G. , & Martinez, G. (2025). Key facts about U.S. Latinos. Pew Research Center. https://www.pewresearch.org/short‐reads/2025/10/22/key‐facts‐about‐us‐latinos/

[jora70232-bib-0041] Quezada, C. , & Fuentes, F. (2025). Mental health and its importance in supporting Hispanic and Latino students. In A. Villarreal (Ed.), Hispanic scholar perspectives on education and wellbeing (pp. 209–242). IGI Global Scientific Publishing. 10.4018/979-8-3693-7688-1.ch008

[jora70232-bib-0042] Rasmussen, E. E. , Shannon, K. L. , & Pitchford, B. (2022). Adolescents' disclosure of mental illness to parents: Preferences and barriers. Health Communication, 37(3), 346–355. 10.1080/10410236.2020.1839201 33106039

[jora70232-bib-0043] Rössler, W. (2016). The stigma of mental disorders. EMBO Reports, 17(9), 1250–1253. 10.15252/embr.201643041 27470237 PMC5007563

[jora70232-bib-0044] Sampaio, F. , Gonçalves, P. , & Sequeira, C. (2022). Mental health literacy: It is now time to put knowledge into practice. International Journal of Environmental Research and Public Health, 19(12), 7030. 10.3390/ijerph19127030 35742278 PMC9222847

[jora70232-bib-0045] Sanchez, K. , Killian, M. O. , Eghaneyan, B. H. , Cabassa, L. J. , & Trivedi, M. H. (2019). Culturally adapted depression education and engagement in treatment among Hispanics in primary care: Outcomes from a pilot feasibility study. BMC Family Practice, 20(1), 103. 10.1186/s12875-019-1031-7 31638915 PMC6802339

[jora70232-bib-0046] Santacrose, D. E. , Kia‐Keating, M. , & Lucio, D. (2021). A systematic review of sociological factors, community violence exposure, and disparities for Latinx youth. Journal of Traumatic Stress, 4(5), 1027–1044. 10.1002/jts.22733 34647363

[jora70232-bib-0047] Santiago‐Rivera, A. L. (2003). Latinos, value, and family transitions: Practical considerations for counseling. Journal of Counseling and Human Development, 35, 1–12.

[jora70232-bib-0048] Suppiej, A. , Longo, I. , & Pettoello‐Mantovani, M. (2025). The pivotal role of mental health in child and adolescent development. Global Pediatrics, 13, 100277. 10.1016/j.gpeds.2025.100277

[jora70232-bib-0049] Thornicroft, G. (2006). Shunned: Discrimination against people with mental illness. Oxford University Press.

[jora70232-bib-0050] Tien, C. (2023). How the stigma of mental health harms Hispanic adolescents. UC Merced Undergraduate Research Journal, 15, 868. 10.5070/m415160868

[jora70232-bib-0051] UnidosUS . (n.d.). Health. https://unidosus.org/issues/health/

[jora70232-bib-0052] U.S. Census Bureau . (2024). New estimates highlight differences in growth between the U.S. Hispanic and Non‐Hispanic populations. Census.Gov. https://www.census.gov/newsroom/press‐releases/2024/population‐estimates‐characteristics.html

[jora70232-bib-0053] U.S. Department of Health and Human Services, Office of Minority Health . (2025). Mental and behavioral health: Hispanic/Latinos. https://minorityhealth.hhs.gov/mental‐and‐behavioral‐health‐hispanics

[jora70232-bib-0054] Valle, R. , Yamada, A. M. , & Matiella, A. C. (2006). Fotonovelas: A health literacy tool for educating Latino older adults about dementia. Clinical Gerontologist, 30(1), 71–88. 10.1300/J018V30N01_06

[jora70232-bib-0055] Vargas, S. M. , Cabassa, L. J. , Nicasio, A. , De La Cruz, A. A. , Jackson, E. , Rosario, M. , Guarnaccia, P. J. , & Lewis‐Fernández, R. (2015). Toward a cultural adaptation of pharmacotherapy: Latino views of depression and antidepressant therapy. Transcultural Psychiatry, 52(2), 244–273. 10.1177/1363461515574159 25736422

[jora70232-bib-0056] Vega, W. A. , Rodriguez, M. A. , & Ang, A. (2010). Addressing stigma of depression in Latino primary care patients. General Hospital Psychiatry, 32(2), 182–191. 10.1016/j.genhosppsych.2009.10.008 20302993

[jora70232-bib-0057] Villatoro, A. P. , Morales, E. S. , & Mays, V. M. (2014). Family culture in mental health help‐seeking and utilization in a nationally representative sample of Latinos in the United States: The NLAAS. American Journal of Orthopsychiatry, 84(4), 353–363. 10.1037/h0099844 24999521 PMC4194077

[jora70232-bib-0058] Wang, C. , Barlis, J. , Do, K. A. , Chen, J. , & Alami, S. (2019). Barriers to mental health help seeking at school for Asian‐ and Latino/a‐American adolescents. School Mental Health: A Multidisciplinary Research and Practice Journal, 12, 182–194. 10.1007/s12310-019-09344-y

[jora70232-bib-0059] Watkins, D. C. (2017). Rapid and rigorous qualitative data analysis. International Journal of Qualitative Methods, 16(1), 12131. 10.1177/1609406917712131

[jora70232-bib-0060] Wisdom, J. P. , Clarke, G. N. , & Green, C. A. (2006). What teens want: Barriers to seeking care for depression. Administration and Policy in Mental Health and Mental Health Services Research, 33(2), 133–145. 10.1007/s10488-006-0036-4 16489480 PMC3551284

[jora70232-bib-0061] World Health Organization . (2025). Mental health of adolescents. World Health Organization. https://www.who.int/news‐room/fact‐sheets/detail/adolescent‐mental‐health

